# Tumour-draining lymph nodes in head and neck cancer are characterized by accumulation of CTLA-4 and PD-1 expressing Treg cells

**DOI:** 10.1016/j.tranon.2022.101469

**Published:** 2022-06-14

**Authors:** Krzysztof Piersiala, Pedro Farrajota Neves da Silva, Vilma Lagebro, Aeneas Kolev, Magnus Starkhammar, Alexandra Elliot, Linda Marklund, Eva Munck-Wikland, Gregori Margolin, Susanna Kumlien Georén, Lars-Olaf Cardell

**Affiliations:** a Division of ENT Diseases, Department of Clinical Sciences, Intervention and Technology, Karolinska Institutet, Stockholm; b Department of Otorhinolaryngology, Karolinska University Hospital, Stockholm, Sweden; c Department of Pathology and Cytology, Karolinska University Hospital, Stockholm, Sweden; d Medical unit Head Neck, Lung and skin Cancer, Karolinska University Hospital, Stockholm, Sweden

**Keywords:** T regulatory cells, CTLA-4, PD-1, HNSCC, Immunotherapy

## Abstract

**Introduction:**

High Tregs infiltration within the tumour microenvironment (TME) of various cancers shows a positive correlation with poor prognosis. Despite the fact that tumour draining lymph nodes (TDLNs) are recognized as key organs playing a crucial role in response to immunotherapy and modulating anti-cancer immunity, the distribution of Tregs and their role in TDLNs remain uncertain thus far. The purpose of this project is to investigate the density of Tregs in TDLNs and non-TDLNs and their expression of immune checkpoint molecules – PD-1 and CTLA-4.

**Methods:**

Samples including TDLNs, non-TDLNs and metastatic lymph nodes (LNs) from 23 patients with oral squamous cell carcinoma (OSCC) were analyzed by multicolour flow cytometry with a focus on Tregs population and expression of CTLA-4 and PD-1.

**Results:**

TDLNs and metastatic LNs were characterized by a significantly higher infiltration of Tregs defined as CD4+FoxP3+CD25^high^CD127^low^ cells and significantly higher expression of CTLA-4 and PD-1 on Tregs compared with non-TDLNs. Tregs in TDLNs and metastatic LNs co-expressed CTLA-4 and PD-1 abundantly. High expression of these immune check-point molecules correlated with positive N-stage but not with T-stage.

**Conclusion:**

TDLNs and metastatic LNs are characterized by a high accumulation of Tregs expressing high levels of CTLA-4 and PD-1. High infiltration of Tregs can be a potential driver of an immunosuppressive milieu in TDLNs that can, in turn, favour cancer progression. High accumulation of Tregs expressing CTLA-4 and PD-1 in TDLNs is associated with lymph node involvement, but not with the size of the primary tumour.

## Highlights

• Tumour-draining lymph nodes (TDLNs) in HNSCC are characterized by high infiltration of Tregs.
• Tregs in TDLNs express higher levels of CTLA-4 and PD-1 compared with non-TDLNs.
• Expression of CTLA-4 on Tregs correlated with positive N-stage.

## Introduction

Oral squamous cell carcinoma (OSCC) is the most common head and neck cancer and accounts for approximately 380 000 cases and 180 000 deaths worldwide annually [1]. OSCC has had an increasing incidence worldwide with a trend showing a significant increase, especially among younger patients [2]. Despite recent advances in surgical and oncological treatment, the prognosis is still relatively unfavourable with 5-year relative survival of 66,9% [3]. Oral cancer has a deceitful tendency for occult metastases which hampers the prognosis significantly why different techniques to detect and investigate the tumour draining lymph nodes (TDLNs) also known as sentinel node(s) have emerged. Trans Oral Robotic Surgery (TORS) has recently emerged as another advancement aiming at improving the outcomes and de-escalation of the adjuvant treatment [4]. Furthermore, cancer immunotherapy is one of the additional advancements in the management of OSCC. The blockade of immune checkpoint molecules (ICIs) such as programmed cell death protein 1 (PD-1) and cytotoxic T-lymphocyte-associated protein 4 (CTLA-4) on host immune cells showed a remarkable efficacy across different cancer types and significantly improved survival in some patients, especially with advanced stages of the disease [5], [6], [7]. Still, a significant fraction of patients treated with ICIs do not respond to treatment or achieve only stabilization of the disease [8]. Thus, there is a need for the development of reliable biomarkers identifying responders. As ICI modifies the host's immune system, it seems natural to explore in-depth antitumor responses to understand resistance mechanisms.

There is accumulating evidence suggesting that CD4^+^ regulatory T cells (Tregs), which play a crucial role in inhibiting anti-tumour immunity, may be contributing to the clinical failure of ICIs [9]. Tregs are a heterogenous group within CD4^+^ T cells. The major subtype – natural Tregs (nTregs) - are defined by the expression of nuclear transcription factor Foxp3 and high levels of CD25 and low expression of CD127. High Tregs infiltration within the tumour microenvironment (TME) of various cancers showed a positive correlation with poor prognosis [10], [11], [12], [13]. A recent metanalysis by Shang et al. showed that high infiltration with FoxP3 cells correlated with significantly shorter overall survival in majority of solid tumours [14]. The prognostic significance of Tregs infiltration in TME of head and neck cancer remains controversial. While Seminerio et al. reported that Tregs infiltration improved disease-free and overall survival in HNSCC patients, Sun et al. and Badoual et al. reported a positive correlation between Tregs and worse prognosis [15], [16], [17]. Hanakawa et al. showed also that high levels of Tregs (CD4^+^FoxP3^+^ cells) in TME of early-stage OSCC correlated with poor prognosis [18]. Furthermore, Tregs in TME have been shown to express upregulated levels of immune checkpoint molecules such as CTLA-4, PD-1, TIM-3 that can further inhibit the anti-cancer immune response by inhibiting effector T cells and influencing the function of antigen-presenting cells (APCs) [19], [20].

Recent research has been focused on finding predictive biomarkers in the blood or TME. However, the importance of the tumour draining lymph nodes (TDLN) as the source of tumour-specific CD4^+^ and CD8^+^ cells and the site of important anticancer immunological events such as antigen presentation, immune cell activation, priming, proliferation and differentiation needs much more attention. TDLNs are defined as one or a group of first lymph nodes draining a tumour. Non-tumour-draining lymph nodes (n-TDLNs) come from the same patient's neck but do not directly drain the tumour. Even though TDLNs have been largely overlooked in recent literature, there is still accumulating evidence suggesting their pivotal role in orchestrating anti-cancer immune response and them being a site of action for immunotherapies [21], [22], [23], [24]. Deng et al. showed that accumulation of Foxp3+ Tregs in TDLNs is correlated with disease progression and contributed to an immunosuppressive milieu in colorectal cancer [25]. The distribution of Tregs in TDLNs and their expression of immune checkpoint molecules in HNSCC has not been studied before. Thus, this project aims to investigate and describe the Tregs population in tumour draining lymph nodes (TDLNs) alternatively, also called Sentinel Nodes (SNs) and compare them with non-tumour draining lymph nodes (n-TDLNs).

## Materials and methods

### Patient characteristics

Eligible patients enrolled for this study met the following inclusion criteria: 1) diagnosis of primary or recurrent oral cancer squamous cell carcinoma (OSCC), 2) tumour/recurrence excision with sentinel node assisted elective neck dissection (identification in SPECT-CT, and location confirmed intraoperatively by gamma probe and injection of indocyanine green (ICG) and further visualization with near-infrared light) performed at Karolinska University Hospital, Stockholm, Sweden between March 2019 and June 2020, 3) willingness to participate in the study. For details regarding the setting of sentinel node procedure at Karolinska University Hospital, see the paper of Kågedal et al. [26]. Exclusion criteria were as follows: 1) systematic autoimmune diseases 2) synchronous second malignancies, hemo-lymphopoietic malignancies in the past 3) any other acute or chronic condition that could influence immunological milieu in the lymph nodes. The flow chart of study participants is shown in Supplementary Figure 1.

### Sample preparation

The unfixed neck sample after excision was transferred directly to the Pathology Department, where one of the designated pathologists (P.F.S.) handled samples and separated lymph nodes halves (all TDLNs and 1-2 n-TDLNs per one patient). The lymph nodes after surgical excision were kept in pre-chilled MACS Tissue Storage Solution and used within 1 hour for further analysis. Tumour Dissociation KIT (Miltenyi Biotec #130-100-008) was used to dissociate surgical specimens mechanically and enzymatically. After dissociation, cells were filtered through a 100µm cell strainer (BD biosciences #352360). Cells were re-suspended in brilliant stain buffer (BD biosciences #563794) at 40*10*^6 cells/ ml and used for downstream analysis.

### Flow cytometry

Single-cell suspensions with purified cells from blood and surgical specimens were first blocked with Fc-block for 5 minutes at room temperature. Next, samples were stained with an antibody panel (LIVE/DEAD™ Fixable Far Red Dead Cell Stain Kit, CD3, CD4, CD8, CD25, CD127, PD-1, CTLA-4, CD69, HLA-DR,) for 20 minutes at room temperature. Staining was followed by two washing steps performed with PBS, 400g, for 5 minutes. For FoxP3 intracellular staining, cells were fixed and permeabilized using BD Cytofix/Cytoperm™ solution (BD Biosciences, 560098) according to the manufacturer's protocol. For washing steps, Perm Wash Buffer (BD Biosciences, 554723) was used following the manufacturer's protocol. After two additional washes cells were resuspended in PBS with 1% paraformaldehyde (HistoLab #02178) and analyzed on LSR FORTESSA (BD Biosciences). Analysis of the flow cytometry data was performed with FlowJo version 10.8.0 (LLC, USA).

Cells were first gated based on side scatter (SSC-A) and forward scatter (FSC-A) to exclude debris. Based on Live/dead staining with LIVE/DEAD™ Fixable Far Red Dead Cell Stain Kit, viable cells were selected. Cells were then gated manually to delineate following cell subpopulations CD3^+^CD4^+^, CD3+CD4+FoxP3+, CD3+CD4+FoxP3+CTLA-4+, CD3+CD4+FoxP3+PD-1+, CD3+CD4+FoxP3+CD25^high^CD127^low^. Then the expression of CTLA-4 and PD-1 was analysed individually on the aforementioned populations. FMO negative controls were used for CTLA-4 and PD-1 antibodies. Summary of the gating strategy for T regulatory cells is showed in Supplementary Figure 2.

### Statistical analysis

Statistical analyses were performed with GraphPad Prism version 9.0.0 (GraphPad Software, La Jolla, CA, USA). The Kolmogorov-Smirnov normality test was used to determine if data sets were normally distributed, and Mann-Whitney or two-tailed Unpaired t-test were chosen, depending on the distribution of the data. Paired t-test was used to compare paired groups of data. P < 0.05 (*) was considered significant, and P < 0.01 (**), P < 0.001 (***), P < 0.0001 (****) were considered highly significant.

### Ethical approval

All procedures performed in studies involving human participants were in accordance with the ethical standards of the institutional and/or national research committee and with the 1964 Helsinki declaration and its later amendments or comparable ethical standards. Informed consent was obtained from all individual participants included in the study. Regional Ethics Committee Approvals: 2015/1650-31/2 and 2019-03518.

## Results

### Patient clinical and pathological characteristics

Twenty-three patients with OSCC were enrolled in the study. There were 8 female (34,8%) and 15 male (65,2%) patients with the age range of 23 – 82 and mean age of 62.7 ± 16. The summary of clinical and pathological characteristics is shown in Table 1. Patients with known nodal metastases (cN+) were also included. Detailed clinical information is presented in Supplementary Table 1. A total of 46 TDLNs and 22 n-TDLNs were investigated. One patient contributed with one lymph node, seven with two lymph nodes, seven with three lymph nodes and eight with four lymph nodes (both TDLNs and n-TDLNs). Eighteen patients were diagnosed with tongue squamous cell carcinoma and five with the floor of the mouth squamous cell carcinoma. Twelve patients (52,2%) had nodal metastases confirmed in histopathology.

**Table 1:** Summary of the clinical and pathological characteristics of enrolled patients.

Clinical/pathological characteristic	
Age	
Mean (± SD)	62.7 ± 16
	N (%)
Sex	
Female	8 (34,8%)
Male	15 (65,2%)
Smoking history	
Yes	8 (34,8%)
No	15 (65,2%)
Tumour localization	
Tounge	18 (78,3%)
Floor of mouth	5 (21,7%)
T-stage	
T1	11 (47,8%)
T2	8 (34,8%)
T3	4 (17,4%)
N-stage	
N0	11 (47,8%)
N1	6 (26,1%)
N2a-c	6 (26,1%)

### TDLNs and metastatic lymph nodes accumulate a significantly higher percentage of Tregs

**Fig. 1:**
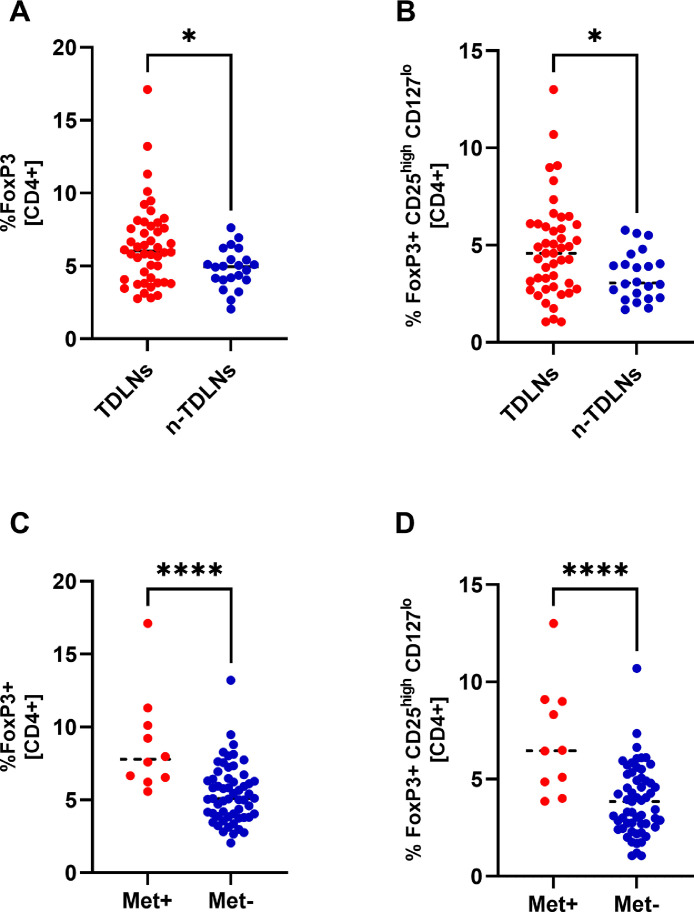
Distribution of FoxP3+ cells and Tregs in TDLNs and n-TDLNs (A-B). Distribution of FoxP3+ cells and Tregs in Met+ LNs and Met- LNs (C-D). *<.05, **<.01, ***<.001, ****<0.0001

In flow cytometry, we identified FoxP3+ positive population defined as CD3+/CD4+/FoxP3+ and T regulatory lymphocytes (Tregs) defined as CD3+/CD4+/FoxP3+/CD25^high^/CD127^low^. The frequency of CD4+FoxP3+ cells and Tregs was significantly higher in TDLNs compared with n-TDLNs (p = 0,0186 and p = 0,0197, respectively) (Fig. 1 A-B). The average percentage of Tregs in TDLNs was 4,8% and 3,4% in n-TDLNs.

Ten lymph nodes, which we investigated, contained metastases verified in histopathology. Those metastatic lymph nodes were characterized by a high accumulation of CD4+FoxP3+ cells and Tregs compared with lymph nodes without metastases. Metastatic lymph nodes contained at average 7.02% Tregs compared with 3.90% in all lymph nodes without metastases (p <0,0001) (Fig. 1C-D).

### Accumulation of CTLA-4 expressing Tregs in TDLNs and metastatic lymph nodes

**Fig. 2:**
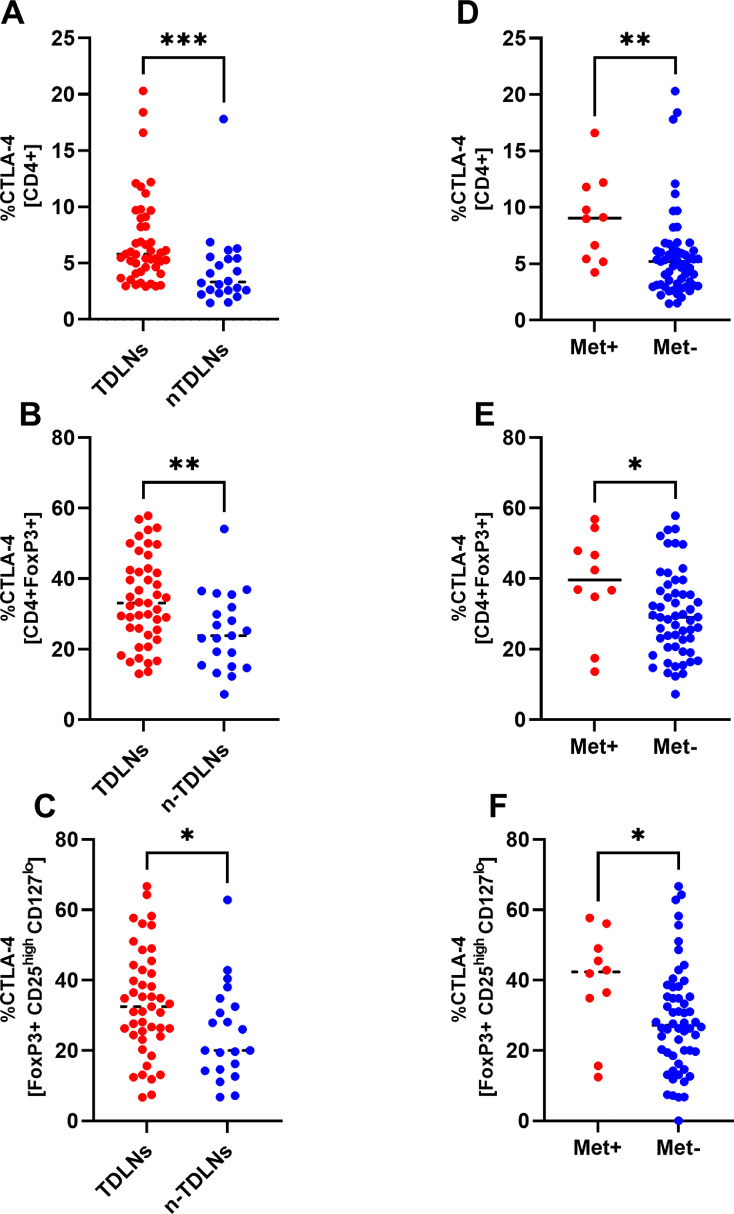

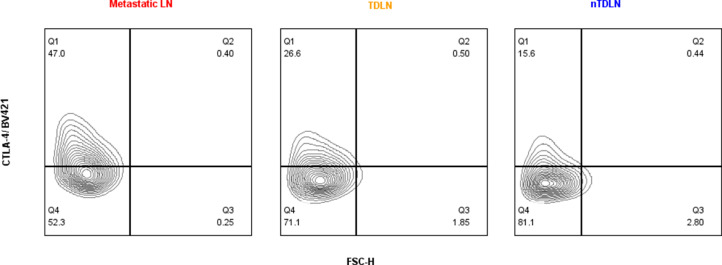
Comparison of TDLNs and n-TDLNs in regard to percentage of CTLA-4+ cells within CD4+ gate (A), CD4+FoxP3+ gate (B) and CD4+FoxP3+CD25^high^CD127^lo^ gate (C). Comparison of Met+ LNs and Met- LNs in regard to percentage of CTLA-4+ cells within CD4+ gate (D), CD4+FoxP3+ gate (E) and CD4+FoxP3+CD25^high^CD127^lo^ gate (F). *<.05, **<.01, ***<.001, ****<0.0001. Fig. 2G. Representative flow cytometry analysis of CTLA-4 expression within CD4+FoxP3+CD25^high^CD127^lo^ gate in Metastatic LN, TDLN and nTDLN.

The flow cytometry analysis revealed that CD4+, CD4+FoxP3+ and Tregs in TDLNs and metastatic lymph nodes (LNs) expressed significantly higher percentage of CTLA-4 as compared with n-TDLNs. (Fig. 2A-F). Representative dot plots of CTLA-4 expression on Tregs are presented in Fig. 2G. In average, CD4+ cells in TDLNs expressed CTLA-4 at 5,80% compared with 3,33% in n-TDLNs (p< 0,0005). The same observation was made in comparison of metastatic (Met+) and non-metastatic lymph nodes (Met-) (9,05% vs. 5,20%, p<0,0047)(Fig. 2A, D). FoxP3 positive CD4+ cells and Tregs expressed CTLA-4 at significantly higher levels (Fig. 2B, C, E, F). Tregs in TDLNs expressed CTLA-4 at 33,58% in average compared with 25,03% in n-TDLNs (p= 0,0313). Tregs in metastatic LNs were characterized by even higher expression of CTLA-4 with mean expression of 39,24% in Met+ nodes and 28,79% in Met- (p=0,0499).

### Tregs in lymph nodes express high levels of PD-1 and the PD-1 expression correlates with the presence of metastases in lymph nodes

**Fig. 3:**
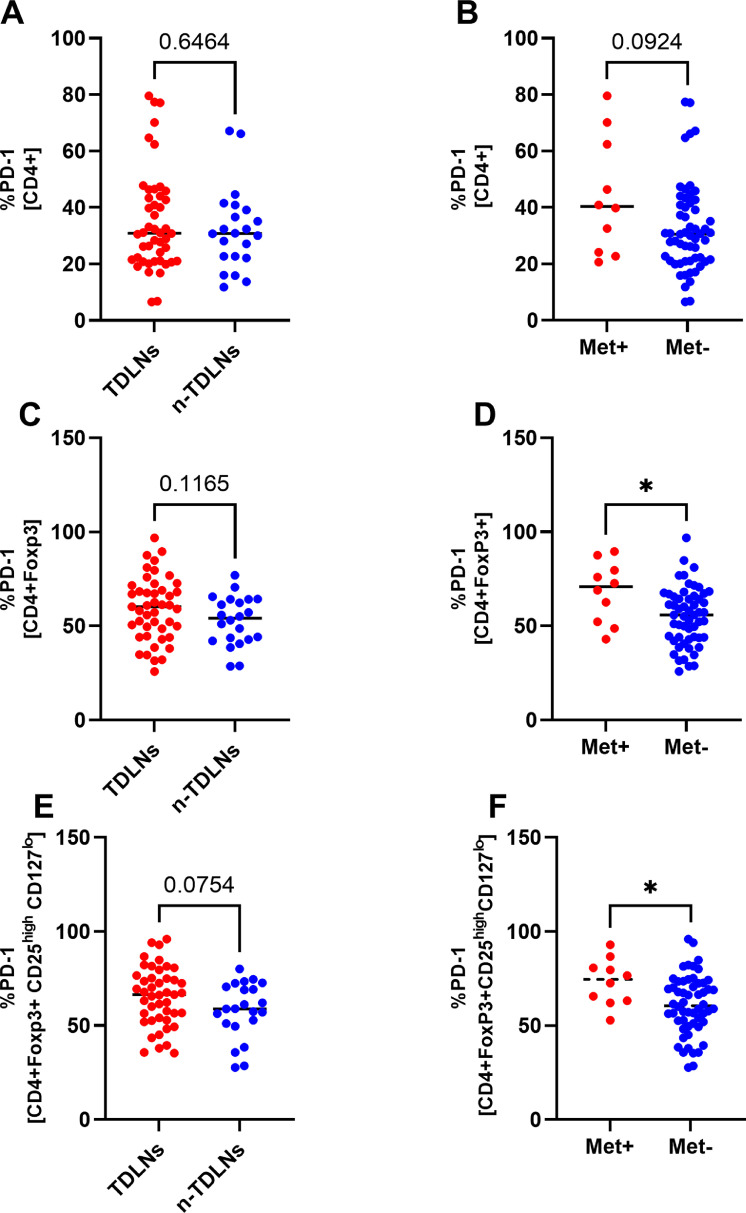
Comparison of TDLNs and n-TDLNs in regard to percentage of PD-1+ cells within CD4+ gate (A), CD4+FoxP3+ gate (B) and CD4+FoxP3+CD25^high^CD127^lo^ gate (C). Comparison of Met+ LNs and Met- LNs in regard to percentage of CTLA-4+ cells within CD4+ gate (D), CD4+FoxP3+ gate (E) and CD4+FoxP3+CD25^high^CD127^lo^ gate (F). *<.05, **<.01, ***<.001, ****<0.0001

CD4+ cells in TDLNs and n-TDLNs express PD-1 at comparable levels with an average percentage of PD-1 positive cells of 30.90% and 30.80%, respectively. Tregs tend to express higher levels of PD-1 on their surface with an average of 65,32% positive cells in TDLNs and 58,06% in n-TDLNs (p=0,0754). High expression of PD-1 correlates with the presence of metastases in lymph nodes. Met+ LNs had significantly higher expression of PD-1 on Tregs and FoxP3+ cells compared with Met- LNs (p= 0,0165 and p= 0,0217, respectively)(Fig. 3A-F).

### Tregs in TDLNs and Metastatic LNs co-express abundantly PD-1 and CTLA-4

**Fig. 4:**
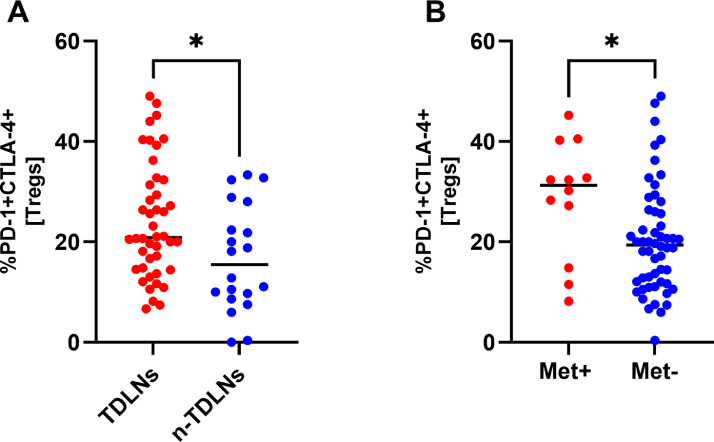

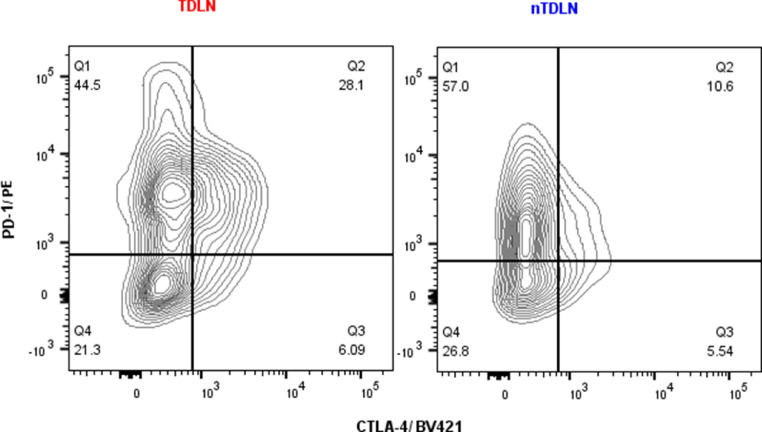
A-B Comparison of TDLNs and n-TDLNs in regard to percentage of CTLA-4+ and PD-1+ cells within CD4+FoxP3+CD25^high^CD127^lo^ gate (A). Comparison of Met+ LNs and Met- LNs in regard to percentage of CTLA-4+ and PD-1+ cells within CD4+FoxP3+CD25^high^CD127^lo^ gate (B) *<.05, **<.01, ***<.001, ****<0.0001. Fig. 4C. Representative flow cytometry analysis of CTLA-4 and PD-1 expression within CD4+FoxP3+CD25^high^CD127^lo^ gate in TDLN and nTDLN.

Multicolour flow cytometry analysis revealed significant numbers of Tregs co-expressing PD-1 and CTLA-4 in the studied compartments. In TDLNs and Met+ LNs, there was a significantly higher fraction of double-positive cells observed compared with n-TDLNs and Met- LNs. In TDLNs, on average 20,85% of Tregs were double-positive, whereas in n-TDLNs 15,45% (p=0,0246)(Fig. 4A). Metastatic LNs were characterized by an even higher rate of double-positive cells with on average 31,3% double positive Tregs compared with 19,4% in Met- LNs (p=0,0243)(Fig. 4B). Representative dot plots of double-positive cells on Tregs are presented in Fig. 4C.

### Expression of CTLA-4 and PD-1 on Tregs correlates with N stage but not with T stage

**Fig. 5:**
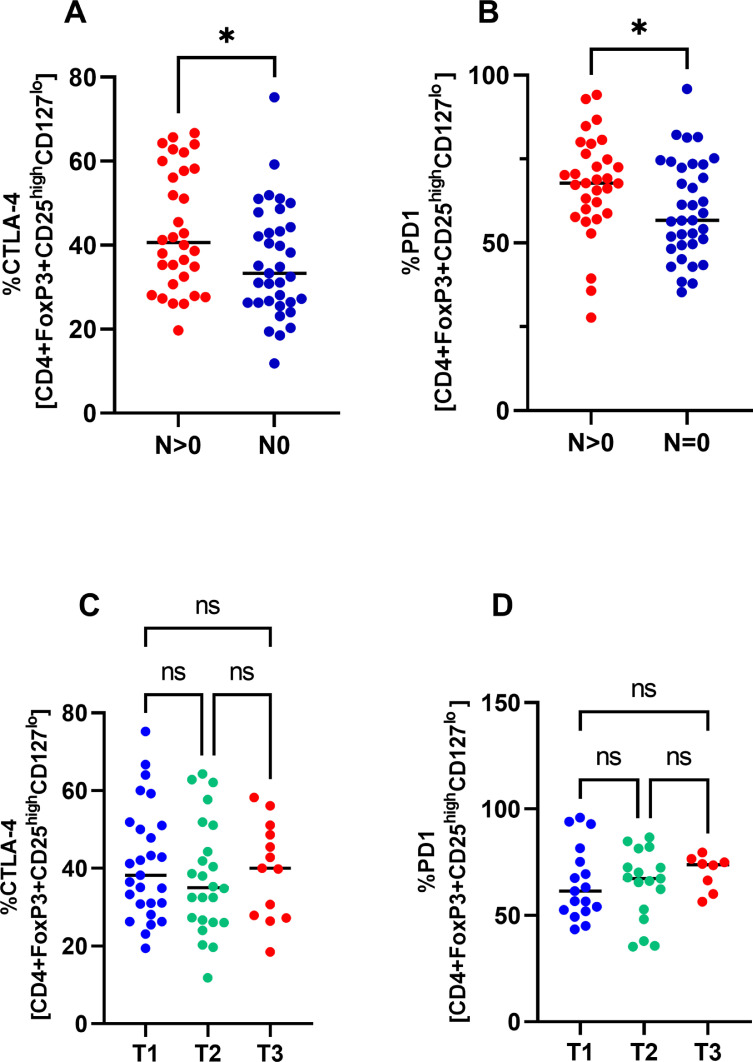
Comparison of percentage CTLA-4 and PD-1 positive Tregs regarding the presence of nodal metastases (N-stage) and size of the primary tumour (T-stage). *<.05

Next, we analyzed the relationship between the expression of CTLA-4 and PD-1 on Tregs and TNM clinical staging. As shown in Fig. 4, expression of CTLA-4 and PD-1 on Tregs was significantly higher in patients with pN+ stage compared with them who had been classified as pN0 (Fig. 5A-B). However, there was no significant difference in the frequency of CTLA-4 or PD-1 positive cells between different T-stages (Fig. 5C-D).

### Paired analysis reveals that TDLNs have a higher accumulation of Tregs and higher expression of CTLA-4 compared with n-TDLNs in one patient

**Fig. 6:**
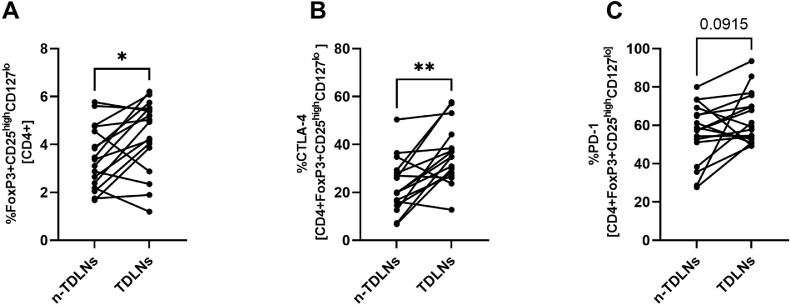
Comparison of the percentage of Tregs (A), expression of CTLA-4 (B) and PD-1(C) between TDLNs and n-TDLNs. All the cases are paired and linked with a line. When more than one TDLN/n-TDLN was obtained per patient, a mean value was calculated and included in the presented analysis.

In order to investigate if levels of Tregs and expression of CTLA-4 and PD-1 differ between patients, we calculated a mean percentage of Tregs, CTLA-4 on Tregs and PD-1 on Tregs in all TDLNs and n-TDLNs from every patient. Then we compared mean values with paired t-test (Fig. 6A-C). The analysis revealed that TDLNs contained a significantly higher percentage of Tregs and higher expression of CTLA-4 on Tregs compared with n-TDLNs (Fig. 6A-C). A similar trend was observed with the expression of PD-1 on Tregs. However, this analysis did not reach statistical significance.

## Discussion

The role of Tregs in anticancer immunity is predominantly studied in TME, where they migrate to and inactivate effector T cells. This immunosuppressive subset of CD4+ cells has recently been associated with resistance to treatment with novel cancer immunotherapies such as anti-CTLA-4 and anti-PD-1 [27]. While many researchers focus on investigating immune cells in TME and peripheral circulation, tumour draining lymph nodes (TDLN) – crucial organs in orchestrating anticancer immunity deserve more attention. Here, we provide the first evidence of the accumulation of FoxP3+ Tregs in TDLNs and metastatic lymph nodes (LNs) in HNSCC. Furthermore, we show that Tregs in these lymph nodes express high levels of CTLA-4 and PD-1 - targets for the ICIs treatment. We observed as well that significant numbers of Tregs in TDLNs and metastatic LNs co-express both CTLA-4 and PD-1. Non-tumour draining lymph nodes (n-TDLNs) in the same patients were characterized by both lower presence of Tregs and lower expression of immune checkpoint molecules.

In HNSCC, there are very few papers investigating immune cells in TDLNs by flow cytometry. Previous projects that we identified used mainly immunohistochemical methods in their investigation [28,29]. However, the Tregs population has previously been investigated in TDLNs in other cancer types such as colorectal cancer, lung cancer, melanoma, gastric, cervical or bladder cancer [25], [30], [31], [32], [33], [34]]. In line with those groups, we confirmed that metastatic LNs in HNSCC are characterized by a high accumulation of FoxP3+ Tregs compared with non-metastatic LNs. In contrast to our findings, Deng et al. reported that the infiltration of Tregs in TDLNs in colorectal cancer was not influenced by the distance of lymph nodes from the primary tumour. In our cohort, TDLNs, also called Sentinel Nodes (SNs), showed significantly higher infiltration of Tregs compared with n-TDLNs that lie further away from the primary tumour. The significance of high infiltration of Tregs in TDLNs and metastatic LNs depend on the contribution of these cells to inducing an immunosuppressive environment in TDLNs, which in turn can lead to cancer immune evasion. Tregs by various mechanisms may suppress anti-tumour immunity. Amongst others, they induce apoptosis and inhibit effector T cells by influencing their activation and proliferation [35]. Furthermore, TDLNs are the site where FoxP3+ Tregs differentiate from naïve CD4+ cells upon antigen presentation in LNs. Differentiated Tregs by expressing chemokine receptors including CCR4, CCR5, CCR10 and others are recruited and trafficked to TME by chemotactic gradient [36,37]. Thus, a high accumulation of Tregs in TDLNs may contribute indirectly to increased immunosuppressive features of TME.

Tregs in TME exhibit highly activated phenotypes with high expression of immune checkpoint molecules such as PD-1, CTLA-4 and TIGIT [38,39]. High expression of those immune checkpoint molecules contributes to inhibition of the interaction between effector T cells and APC cells within TME [40]. Accumulating data suggests that overexpression of immune checkpoint molecules on Tregs in TME favours their survival and support the further expression of suppressive cytokines. While the expression of those molecules on Treg is extensively studied within TME, there is still little known about their expression pattern on Tregs in TDLNs. Van de Ven et al. showed higher expression levels of PD-1 on T cells in TDLNs in lung cancer compared with n-TDLNs [41]. Two other preclinical studies confirmed that PD-1 and PD-L1 are expressed on T cells more abundantly in TDLNs compared with n-TDLNs [21,22]. In our previous study [24], we showed also that T cells in TDLNs from patients with HNSCC express significant levels of PD-1. However, the expression of PD-1 and CTLA-4 on Tregs were not investigated before this project. Here, we show that Tregs in TDLNs and metastatic LNs express abundantly CTLA-4 and PD-1 and thus, these cells most likely interact with anti-CTLA4 and anti-PD-1 antibodies during systemic ICIs treatment. How does a systemic treatment with ICIs influence their function and phenotype remains to be investigated.

The role of TDLNs in cancer immunity has been given increasing attention and is recognized as having a key function in response to immunotherapy. It is now hypothesized that the ICI efficacy relies predominantly on an influx of newly primed T cells from TDLNs into TME, rather than reinvigorating exhausted and fully differentiated T cells infiltrating tumours [42,43]. Based on this assumption, there are at least several ongoing clinical trials, where ICI agents are being given locally into TDLNs. Local treatment targeting TDLNs could be applied not only to patients with advanced disease. Patients with early-stage cancer could also benefit from local application of ICI agents into TDLNs by reducing the risk of distant metastases or recurrence and by enhancing the natural anti-tumour T cell repertoire [43]. Our project is a first step supporting the rationale for local use of immunotherapies in TDLNs in HNSCC. We confirmed that TDLNs in HNSCC are characterized by an immunosuppressive milieu with an accumulation of Tregs with abundant expression of immune checkpoint molecules that can be reversed by ICIs.

## Conclusions

In conclusion, the accumulation of Tregs with high expression of CTLA-4 and PD-1 in TDLNs and metastatic LNs in HNSCC showed by our study suggests that TDLNs are characterized by an immunosuppressive milieu that can, in turn, favour cancer progression. High expression of immune checkpoint molecules on Tregs in TDLNs of patients with early stages of HNSCC justifies clinical trials investigating the use of immunotherapies in early stages of HNSCC or/and local administration of ICI agents into TDLNs that could potentially improve anti-cancer immunity and prevent tumour invasion and spread.

## Author contribution statement

Krzysztof Piersiala – Conceptualization, Data curation, Formal analysis, Investigation, Methodology, Project administration, Writing – original draft. Pedro Farrajota Neves da Silva - Data curation, Investigation, Methodology, Writing – review & editing. Vilma Lagebro - Data curation, Investigation, Project administration, Writing – original draft, Writing – review & editing. Aeneas Kolev – Investigation, Methodology, Project administration, Writing – review & editing. Magnus Starkhammar – Investigation, Methodology, Writing – review & editing. Alexandra Elliot – Investigation, Methodology, Project administration, Writing – review & editing. Linda Marklund – Investigation, Methodology, Project administration, Writing – review & editing. Eva Munck-Wikland – Conceptualization, Supervision, Writing – review & editing. Gregori Margolin – Conceptualization, Investigation, Methodology, Project administration, Writing – review & editing. Susanna Kumlien Georén – Conceptualization, Formal analysis, Investigation, Project administration, Supervision, Writing – original draft, Writing – review & editing. Lars-Olaf Cardell – Conceptualization, Funding acquisition, Supervision, Writing – review & editing.

## Declaration of Competing Interest

The authors declare that they have no known competing financial interests or personal relationships that could have appeared to influence the work reported in this paper.

## Appendix Supplementary materials

Image, application 1

Image, application 2

Image, application 3

## References

[1] Global Cancer Observatory (2022).

[2] Ng J.H. (2017). Changing epidemiology of oral squamous cell carcinoma of the tongue: a global study. Head Neck,.

[3] Surveillance, Epidemiology, and End Results (2021). Sub (1975-2018) - Linked To County Attributes - Time Dependent (1990-2018) Income/Rurality, 1969-2019 Counties.

[4] Meccariello G. (2022). Neck dissection and trans oral robotic surgery for oropharyngeal squamous cell carcinoma. Auris Nasus Larynx.

[5] Hodi F.S. (2010). Improved survival with ipilimumab in patients with metastatic melanoma. N. Engl. J. Med..

[6] Brahmer J. (2015). Nivolumab versus docetaxel in advanced squamous-cell non-small-cell lung cancer. N. Engl. J. Med..

[7] Ferris R.L. (2016). Nivolumab for recurrent squamous-cell carcinoma of the head and neck. N. Engl. J. Med..

[8] Topalian S.L. (2014). Survival, durable tumor remission, and long-term safety in patients with advanced melanoma receiving nivolumab. J. Clin. Oncol..

[9] Haratani K. (2017). Tumor immune microenvironment and nivolumab efficacy in EGFR mutation-positive non-small-cell lung cancer based on T790M status after disease progression during EGFR-TKI treatment. Ann. Oncol..

[10] Liotta F. (2011). Frequency of regulatory T cells in peripheral blood and in tumour-infiltrating lymphocytes correlates with poor prognosis in renal cell carcinoma. BJU Int..

[11] O'Callaghan D.S. (2015). Tumour islet Foxp3^+^T-cell infiltration predicts poor outcome in nonsmall cell lung cancer. Eur. Respir. J..

[12] Li F. (2019). CD4/CD8 + T cells, DC subsets, Foxp3, and IDO expression are predictive indictors of gastric cancer prognosis. Cancer Med..

[13] Miller A.M. (2006). CD4^+^CD25^high^T cells are enriched in the tumor and peripheral blood of prostate cancer patients. J. Immunol..

[14] Shang B. (2015). Prognostic value of tumor-infiltrating FoxP3+ regulatory T cells in cancers: a systematic review and meta-analysis. Sci. Rep..

[15] Seminerio I. (2019). Infiltration of FoxP3+ Regulatory T Cells is a Strong and Independent Prognostic Factor in Head and Neck Squamous Cell Carcinoma. Cancers.

[16] Sun D.-s. (2012). The correlation between tumor-infiltrating Foxp3+ regulatory T cells and cyclooxygenase-2 expression and their association with recurrence in resected head and neck cancers. Med. Oncol..

[17] Badoual C. (2006). Prognostic Value of Tumor-Infiltrating CD4^+^T-Cell Subpopulations in Head and Neck Cancers. Clin. Cancer Res..

[18] Hanakawa H. (2014). Regulatory T-cell infiltration in tongue squamous cell carcinoma. Acta Otolaryngol..

[19] Oweida A. (2018). Resistance to Radiotherapy and PD-L1 Blockade Is Mediated by TIM-3 Upregulation and Regulatory T-Cell Infiltration. Clin. Cancer Res..

[20] Nair V Sasidharan, Elkord E. (2018). Immune checkpoint inhibitors in cancer therapy: a focus on T-regulatory cells. Immunol. Cell Biol..

[21] Fransen M.F. (2018). Tumor-draining lymph nodes are pivotal in PD-1/PD-L1 checkpoint therapy. JCI Insight.

[22] Dammeijer F. (2020). The PD-1/PD-L1-Checkpoint Restrains T cell Immunity in Tumor-Draining Lymph Nodes. Cancer Cell.

[23] Francis D.M. (2020). Blockade of immune checkpoints in lymph nodes through locoregional delivery augments cancer immunotherapy. Sci. Transl. Med..

[24] Piersiala K. (2021). CD4(+) and CD8(+) T cells in sentinel nodes exhibit distinct pattern of PD-1, CD69, and HLA-DR expression compared to tumor tissue in oral squamous cell carcinoma. Cancer Sci..

[25] Deng L. (2010). Accumulation of Foxp3^+^T regulatory cells in draining lymph nodes correlates with disease progression and immune suppression in colorectal cancer patients. Clin. Cancer Res..

[26] Kågedal Å. (2020). A novel sentinel lymph node approach in oral squamous cell carcinoma. Curr. Pharm. Des..

[27] Kumagai S. (2020). The PD-1 expression balance between effector and regulatory T cells predicts the clinical efficacy of PD-1 blockade therapies. Nat. Immunol..

[28] Pretscher D. (2009). Distribution of immune cells in head and neck cancer: CD8+ T-cells and CD20+ B-cells in metastatic lymph nodes are associated with favourable outcome in patients with oro- and hypopharyngeal carcinoma. BMC Cancer.

[29] Russell S. (2013). Immune cell infiltration patterns and survival in head and neck squamous cell carcinoma. Head Neck Oncol..

[30] Schneider T. (2011). Foxp3(+) regulatory T cells and natural killer cells distinctly infiltrate primary tumors and draining lymph nodes in pulmonary adenocarcinoma. J. Thorac. Oncol..

[31] Faghih Z. (2016). CD8+ T Lymphocyte Subsets in Bladder Tumor Draining Lymph Nodes. Iran J Immunol.

[32] Heeren A.M. (2019). Efficacy of PD-1 blockade in cervical cancer is related to a CD8(+)FoxP3(+)CD25(+) T-cell subset with operational effector functions despite high immune checkpoint levels. J. Immunother. Cancer.

[33] van den Hout M. (2017). Melanoma Sequentially Suppresses Different DC Subsets in the Sentinel Lymph Node, Affecting Disease Spread and Recurrence. Cancer Immunol. Res..

[34] Kawaida H. (2005). Distribution of CD4(+)CD25^high^regulatory T-cells in tumor-draining lymph nodes in patients with gastric cancer. J. Surg. Res..

[35] Saleh R., Elkord E. (2020). Acquired resistance to cancer immunotherapy: Role of tumor-mediated immunosuppression. Semin. Cancer Biol..

[36] Deng G. (2018). Tumor-infiltrating regulatory T cells: origins and features. Am J Clin Exp Immunol.

[37] Gobert M. (2009). Regulatory T cells recruited through CCL22/CCR4 are selectively activated in lymphoid infiltrates surrounding primary breast tumors and lead to an adverse clinical outcome. Cancer Res..

[38] Nishikawa H., Sakaguchi S. (2010). Regulatory T cells in tumor immunity. Int. J. Cancer.

[39] Saleh R., Elkord E. (2019). Treg-mediated acquired resistance to immune checkpoint inhibitors. Cancer Lett..

[40] Keir M.E. (2008). PD-1 and its ligands in tolerance and immunity. Annu. Rev. Immunol..

[41] van de Ven R. (2017). High PD-1 expression on regulatory and effector T-cells in lung cancer draining lymph nodes. ERJ Open Res..

[42] Swartz M.A., Lund A.W. (2012). Lymphatic and interstitial flow in the tumour microenvironment: linking mechanobiology with immunity. Nat. Rev. Cancer.

[43] Rotman J. (2019). Unlocking the therapeutic potential of primary tumor-draining lymph nodes. Cancer Immunol. Immunother..

