# Supplementary figures and images for: Tumour-draining lymph nodes in head and neck cancer are characterized by accumulation of CTLA-4 and PD-1 expressing Treg cells
Source: Transl Oncol. 2022 Jun 14;23:101469. doi: 10.1016/j.tranon.2022.101469 (PMC9207719; doi:10.1016/j.tranon.2022.101469)

Supplementary Figure 1. The flow chart of study participants.

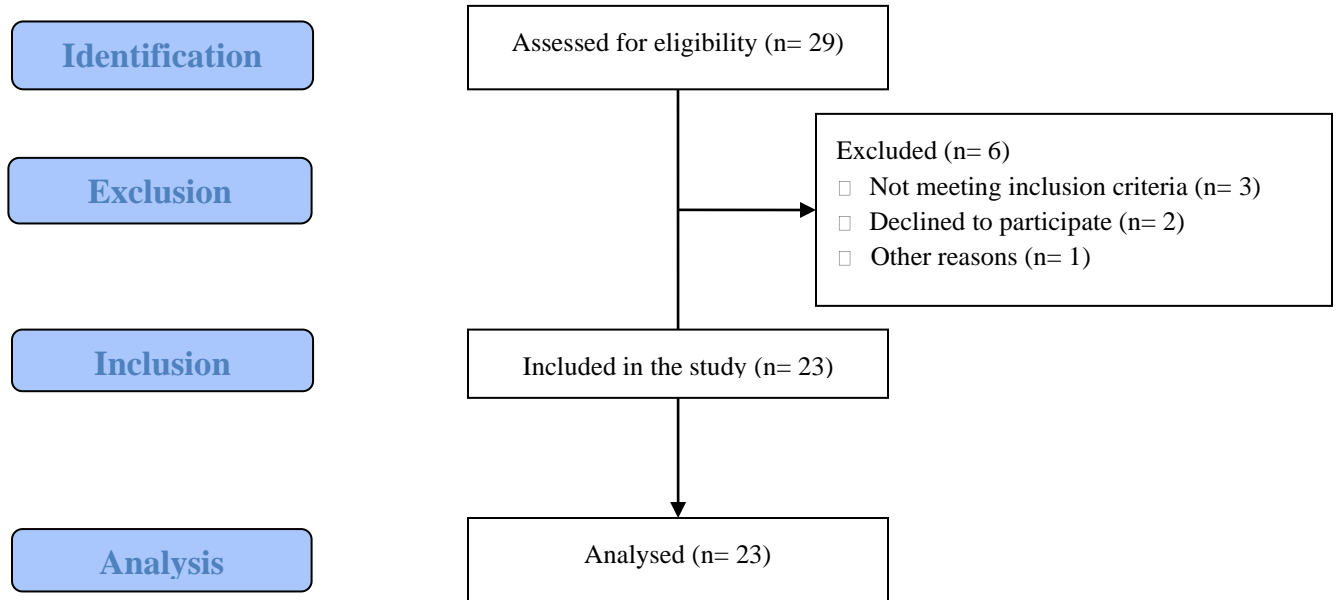

Supplement: Supplementary file 1 [file mmc1.pdf]

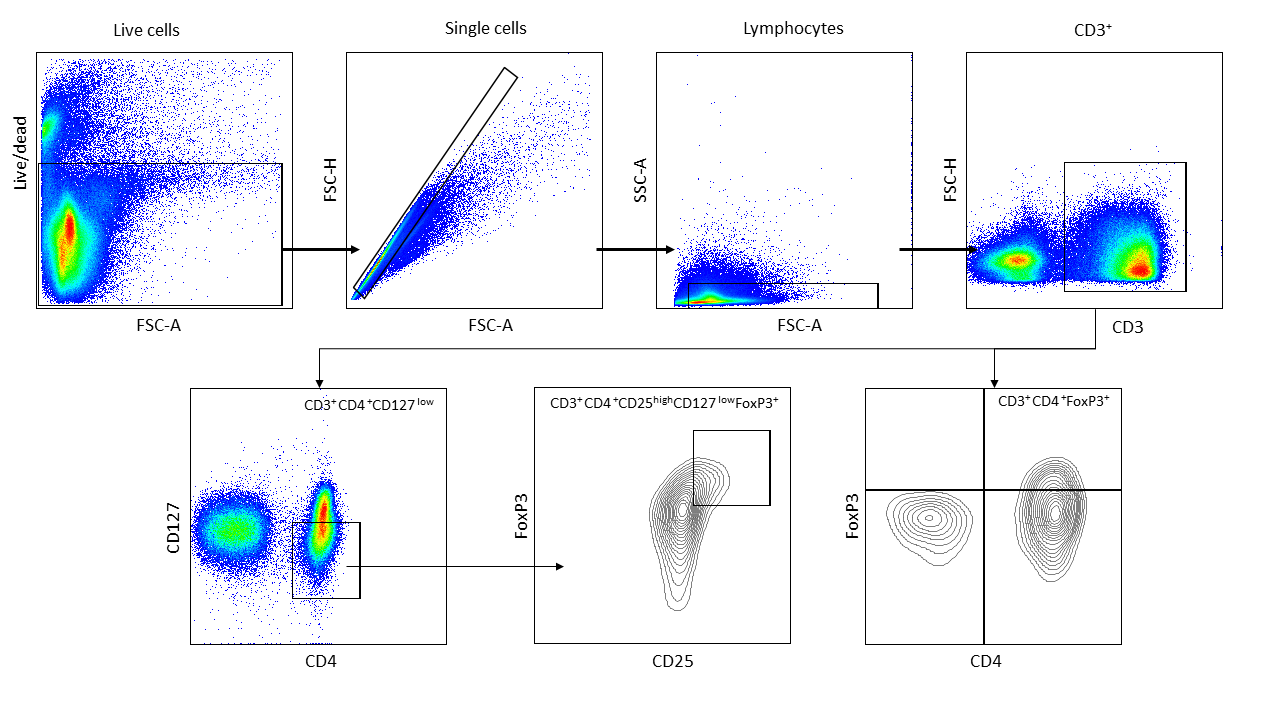

Supplement: Supplementary file 2 [file mmc2.zip › mmc2.tif]
